# Association between shift work and insulin resistance in women: Implications for metabolic health

**DOI:** 10.1371/journal.pone.0337057

**Published:** 2025-11-26

**Authors:** You-Jung Choi, Soo Hyeon Cho, Ja-Ho Leigh, Sung Hoon Jeong

**Affiliations:** 1 Department of Nursing, Seojeong University, Yangju, Korea; 2 Division of Infectious Disease Response, Capital Regional Center for Disease Control and Prevention, Korea Disease Control and Prevention Agency, Seoul, Korea; 3 Department of Nursing, Graduate School of Yonsei University, Seoul, Korea; 4 Department of Rehabilitation Medicine, Seoul National University Hospital, Seoul, Republic of Korea; 5 National Traffic Injury Rehabilitation Research Institute, National Traffic Injury Rehabilitation Hospital, Yangpyeong, Republic of Korea; 6 Institute of Health Policy and Management, Medical Research Center, Seoul National University, Seoul, Korea; 7 Department of Health Policy and Management, Inje University, Gimhae, Korea; Xi’an Jiaotong University, CHINA

## Abstract

Shift work has been associated with circadian rhythm disruption and related metabolic disturbances, with women potentially being more vulnerable due to physiological and hormonal characteristics. Research on the correlation between shift work and insulin resistance in women outside hospital nursing settings remains scarce. Therefore, this study investigated the relationship between shift work and insulin resistance in working-age women, using the triglyceride-glucose (TyG) index as a surrogate marker. Data from 3,780 female participants aged 19–64 years were collected from the 2019–2021 Korea National Health and Nutrition Examination Survey. Participants were classified as day or shift workers, and insulin resistance was categorized as high or low based on the TyG index. The association between work schedule and insulin resistance was evaluated after adjusting for potential confounders. Shift workers had 1.30 times higher odds of elevated insulin resistance than day workers. Stronger associations were observed among women aged 40–50 years, those in pink-collar occupations, and individuals with adverse lifestyle factors, including overweight status, physical inactivity, alcohol consumption, and smoking. These findings underscore the importance of tailored workplace health interventions and the adoption of personalized, circadian rhythm–aligned strategies to reduce metabolic risk among female shift workers, thereby supporting occupational health policy and preventive care.

## Introduction

Shift work—defined by the International Labour Organization as a work schedule that falls outside the standard daytime hours (typically 7:00–8:00 AM to 5:00–6:00 PM)—has become increasingly prevalent worldwide, particularly with the expansion of the service, manufacturing, and healthcare sectors [1]. In Korea, the proportion of workers engaged in shift work is estimated to range between 11.6% and 13.9%, with a growing trend among women, especially in occupations requiring 24-h staffing [2,3]. Although shift work provides operational flexibility and continuity, it is consistently associated with adverse health outcomes, including obesity, diabetes, and cardiovascular disease [4–6]. A substantial body of evidence links shift work to circadian misalignment, a physiological disruption that alters hormonal rhythms, metabolism, and sleep-wake cycles [6].

Circadian rhythm disruption—regulated by the suprachiasmatic nucleus—has been associated with impaired glucose metabolism and reduced insulin sensitivity, which may contribute to insulin resistance. Insulin resistance is a central mechanism in metabolic syndrome and is commonly assessed using surrogate markers due to the limited feasibility of gold-standard tests, such as the hyperinsulinemic-euglycemic clamp [7]. The triglyceride-glucose (TyG) index, derived from fasting glucose and triglyceride levels, has emerged as a practical and reliable surrogate demonstrating high diagnostic accuracy and clinical applicability with up to 96% sensitivity and 99% specificity [8,9]. Recent studies have shown that the TyG index is a stronger predictor of insulin resistance and cardiovascular risk in women than in men [10,11], underscoring its practicality and accessibility as a screening tool derived from routine clinical parameters.

Several previous studies have examined sex differences in the health impacts of shift work, with some reporting that women may be more vulnerable to its adverse metabolic consequences due to hormonal and physiological characteristics [12–14]. Circadian disruption associated with shift work may increase estrogen levels through suppressed melatonin secretion, potentially contributing to a higher prevalence of hormone-related disorders and reproductive health issues among women [15]. Moreover, studies have reported that female shift workers are at a significantly greater risk of metabolic syndrome components, with some studies indicating up to a 6.3-fold higher risk than that for day workers [16–18]. However, much of the existing literature has focused on specific populations, such as healthcare workers or on male-dominated industries like shipbuilding, electronics manufacturing, and transportation [18–20]. Although prior studies have assessed the health impacts of shift work among female hospital nurses [3,17,21], research examining its association with insulin resistance across diverse occupational settings in women remains limited—despite growing evidence of sex-specific metabolic vulnerability.

This study aimed to analyze the differences in insulin resistance according to work patterns among Korean women aged 19–64 years using nationally representative data from the 8th Korea National Health and Nutrition Examination Survey (KNHANES). This age range represents a critical period during which women may be more vulnerable to occupational and behavioral risk factors that influence metabolic health [22]. Despite increasing female participation in shift-based occupations, research on insulin resistance among working-age women across diverse industries remains limited. Given that insulin resistance is a precursor to metabolic syndrome, Type 2 diabetes, and cardiovascular disease, early identification of at-risk populations is essential for long-term prevention and intervention efforts. The TyG index, which can be calculated using routine health screening parameters, has demonstrated high validity as a predictor of insulin resistance in women and offers a simple, cost-effective tool suitable for clinical and occupational health settings [9]. In this study, the TyG index was used as the primary measure of insulin resistance. Therefore, this study provides timely evidence to support early metabolic health screening and inform occupational health strategies tailored to female shift workers.

## Materials and methods

### Study design and participants

The present study was based on a secondary analysis of the KNHANES-VIII dataset spanning 2019–2021 (8th cycle). KNHANES, initiated in 1998, is conducted annually as a nationwide, population-representative cross-sectional survey overseen by the Korea Disease Control and Prevention Agency within the Ministry of Health and Welfare. This survey operates under Article 16 of the National Health Promotion Act to monitor and evaluate the health and nutritional status of the Korean population [23].

**Fig 1:**
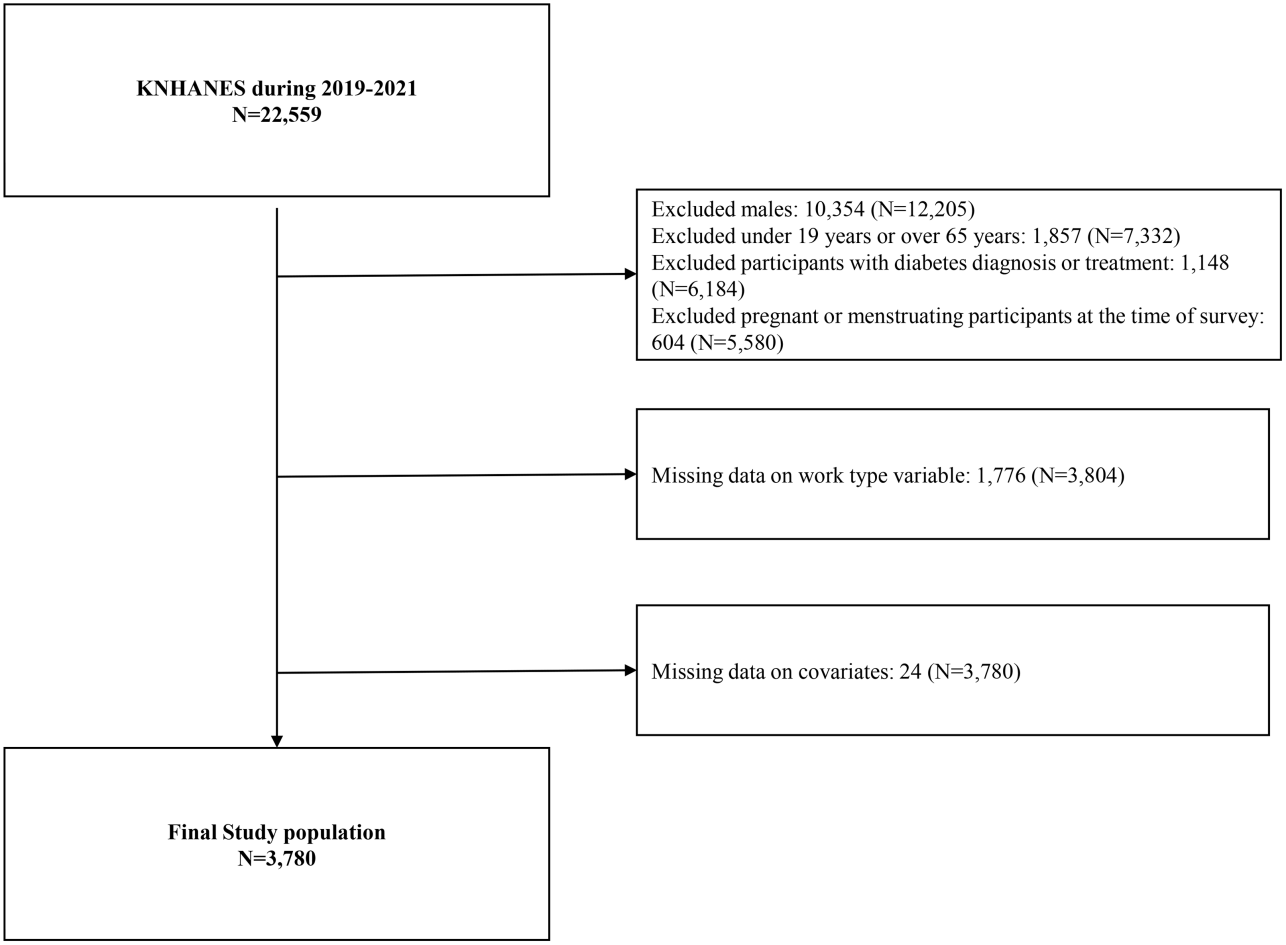
Schematic diagram of the study population.

Of the 22,559 respondents in KNHANES-VIII (2019–2021), 10,354 male participants were excluded to restrict the analysis to women. Next, 1,857 individuals younger than 19 years or aged 65 years and older were excluded to limit the analysis to the working-age population (19–64 years). To avoid confounding in insulin resistance–related analyses, 1,148 participants meeting any diabetes criteria—physician-diagnosed diabetes, use of glucose-lowering medication or insulin, fasting plasma glucose ≥126.0 mg/dL, or HbA1c ≥ 6.5% (48 mmol/mol)—were excluded. Additionally, 604 women who were either pregnant or menstruating at the time of the survey were excluded to minimize hormonal variability in biomarker assessments. Furthermore, 1,776 participants with missing work type information and 24 participants with missing data on other covariates were excluded. The final analytic sample comprised 3,780 female participants (Fig 1).

### Variables

#### Dependent variable.

The primary outcome in this analysis was the TyG index, which combines fasting triglyceride and glucose concentrations and is widely recognized as a surrogate marker of insulin resistance [7]. In KNHANES, blood specimens were obtained after an overnight fast beginning at 19:00 on the day before the examination. The TyG index was calculated as the natural logarithm of [triglycerides (mg/dL) × fasting plasma glucose (mg/dL)/ 2] and was reported on a logarithmic scale [24].

#### Independent variable.

The main independent variable was work type, classified as day workers and shift workers. The National Institute for Occupational Safety and Health (NIOSH) defines shift work as any type of work schedule outside regular day work hours [7:00 AM to 6:00 PM] [25]. In KNHANES, work schedule is recorded as one of the following: day work; evening work (14:00–24:00); night work (21:00–08:00 next day); regular rotating shift work (predetermined alternation between day and night shifts); 24-h rotating shift work; split shift work (two or more distinct working periods within a single day); and irregular shift work. Consistent with previous studies using this dataset, participants reporting day work were designated as “day workers,” whereas those in any other category were classified as “shift workers” [26].

#### Control variables.

We adjusted for a comprehensive set of socioeconomic and health-related covariates to minimize confounding [2,27]. Socioeconomic factors included age (categorized as 19–29, 30–39, 40–49, 50–59, 60–69, and ≥70 years), marital status (married vs. single/divorced/widowed/separated), educational attainment (middle school or less, high school, college or higher), household income (low, mid-low, mid-high, high), and occupation (white collar, pink collar, or blue collar). Health-related covariates included body mass index (BMI) (underweight/normal [<25 kg/m²] vs. overweight [≥25 kg/m²]), drinking status (yes vs. no), walking frequency (adequate vs. inadequate), smoking status (never, former, current), total calorie intake (adequate vs. inadequate), and presence of chronic diseases (yes vs. no). These variables were incorporated into multivariable models assessing the relationship between work schedule and the primary outcome.

### Statistical analysis

All statistical calculations incorporated the sample weights assigned to study participants. These weights were provided by KNHANES to yield estimates representative of the Korean population by accounting for the complex survey design and potential nonresponse [23]. Prior to the analyses, participants were classified into insulin resistance groups according to the median TyG index (8.35): the low insulin resistance group (<8.35) and the high insulin resistance group (≥8.35). Insulin resistance cutoffs may vary slightly depending on sample characteristics, and previous studies conducted in Korea have suggested optimal cutoff values in the range of 8.3–8.5 [28–30]. To determine a valid cutoff value for this study, the TyG index was further evaluated using a receiver operating characteristic curve for impaired fasting glucose. This analysis yielded an optimal threshold of 8.3509. Therefore, a TyG index of 8.35 was deemed an appropriate threshold for differentiating between low and high insulin resistance. We first evaluated the association of work type, socioeconomic and health-related variables, and survey year with insulin resistance using univariate linear regression analyses. Next, multivariable linear regression analyses—adjusting for relevant covariates—were conducted to assess the relationship between work type and insulin resistance. Furthermore, subgroup analyses were performed by fitting stratified multivariable linear regression models within strata of education level, occupational category, and presence of chronic diseases to examine the association between work type and insulin resistance within each subgroup. To assess the odds of high insulin resistance (TyG ≥ 8.35) by work type, weighted logistic regression models were used, and odds ratios (ORs) with 95% confidence intervals (CIs) were calculated comparing shift work to day work. All statistical analyses were performed using SAS software, version 9.4 (SAS Institute Inc.). Statistical significance was defined as a two-sided p-value <0.05.

### Ethical approval

This study was exempted from review by the Institutional Review Board of the Korea Disease Control and Prevention Agency (KDCA; identifier: KDCA-2025-05-07) because it used de-identified data from a national epidemiological survey, posed minimal risk to participants, and served the public health interests. The requirement for informed consent was waived due to the retrospective nature of the study. All procedures were conducted in accordance with the guidelines of the KNHANES.

## Results

Table 1 presents the general characteristics of the study population. Of the 3,780 participants, 3,157 (83.5%) were in the day work group and 623 (16.5%) were in the shift work group. The insulin resistance groups differed significantly for all factors except smoking status, total kcal intake, and survey year.

**Table 1:** General characteristics of the study population.

Variables	Triglycerides and glucose (TyG)						
	Total		Low IR group (%) (<8.5)		High IR group (%) (≥8.5)		p-value
	N	%	N	%	N	%	
Total	3,780	100.0	2,121	56.1	1,659	43.9	
Work type							0.0239
Day work	3,157	83.5	1,797	56.9	1,360	43.1	
Shift work	623	16.5	324	52.0	299	48.0	
Age (years)							<.0001
19–29	696	18.4	512	73.6	184	26.4	
30–39	667	17.6	444	66.6	223	33.4	
40–49	943	24.9	546	57.9	397	42.1	
50–59	1,055	27.9	456	43.2	599	56.8	
60–64	419	11.1	163	38.9	256	61.1	
Marital Status							<.0001
Married	2,422	64.1	1,272	52.5	1,150	47.5	
Single, widow, divorced, separated	1,358	35.9	849	62.5	509	37.5	
Educational level							<.0001
Middle school or less	446	11.8	164	36.8	282	63.2	
High school	1,461	38.7	777	53.2	684	46.8	
College or over	1,873	49.6	1,180	63.0	693	37.0	
Household income							0.0045
Low	254	6.7	133	52.4	121	47.6	
Mid-low	813	21.5	436	53.6	377	46.4	
Mid-high	1,192	31.5	645	54.1	547	45.9	
High	1,521	40.2	907	59.6	614	40.4	
Occupational category ^a^							<.0001
White-collar	2,034	53.8	1,245	61.2	789	38.8	
Pink-collar	1,100	29.1	574	52.2	526	47.8	
Blue-collar	646	17.1	302	46.7	344	53.3	
BMI ^b^							<.0001
Underweight or Normal (<25)	2,833	74.9	1,802	63.6	1,031	36.4	
Overweight (≥25.0)	947	25.1	319	33.7	628	66.3	
Drinking status							0.0002
No	904	23.9	459	50.8	445	49.2	
Yes	2,876	76.1	1,662	57.8	1,214	42.2	
Walking frequency ^c^							<.0001
Inadequate	2,113	55.9	1,105	52.3	1,008	47.7	
Adequate	1,667	44.1	1,016	60.9	651	39.1	
Smoking Status							0.1116
Never	3,220	85.2	1,822	56.6	1,398	43.4	
Former	324	8.6	182	56.2	142	43.8	
Current	236	6.2	117	49.6	119	50.4	
Total kcal intake ^d^							0.5942
Inadequate	2,796	74.0	1,576	56.4	1,220	43.6	
Adequate	984	26.0	545	55.4	439	44.6	
Chronic disease ^e^							<.0001
No	3,140	83.1	1,869	59.5	1,271	40.5	
Yes	640	16.9	252	39.4	388	60.6	
Survey year							0.8425
2019	1,375	36.4	763	55.5	612	44.5	
2020	1,243	32.9	701	56.4	542	43.6	
2021	1,162	30.7	657	56.5	505	43.5	

^a^Occupational categories were classified into white-collar, pink-collar, and blue-collar groups based on the International Standard Classification of Occupations codes. The “inoccupation” group includes homemakers.

^b^BMI: body mass index; obesity status was defined according to the 2018 Clinical Practice Guidelines for Overweight and Obesity in Korea.

^c^Walking frequency: determined based on the recommended walking activity in the Korean Physical Activity Guidelines.

^d^Total kcal intake was calculated as (carbohydrate (g) × 4 kcal/g) + (protein (g) × 4 kcal/g) + (fat (g) × 9 kcal/g).

^e^Chronic disease: included diagnosed diseases such as hypertension and dyslipidemia.

Table 2 presents the association between work type and insulin resistance, adjusted for potential confounders. In the fully adjusted model, shift work was independently and significantly associated with an elevated TyG index compared to day work (adjusted OR = 1.30; 95% CI, 1.08–1.57).

**Table 2:** Association between work type and the triglyceride-glucose index.

Variables	TyG index (≥8.5)	
	Adjusted OR*	95% CI
**Work type**		
Day work	1.00	
Shift work	1.30	(1.08–1.57)
**Age (years)**		
19–29	1.00	
30–39	1.41	(1.08–1.83)
40–49	1.95	(1.51–2.51)
50–59	3.21	(2.47–4.17)
60–64	3.08	(2.20–4.33)
**Marital Status**		
Married	1.00	
Single, widow, divorced, separated	1.00	(0.84–1.19)
**Educational level**		
Middle school or less	1.00	
High school	0.80	(0.62–1.04)
College or over	0.72	(0.54–0.97)
**Household income**		
Low	1.00	
Mid-low	1.05	(0.78–1.43)
Mid-high	1.11	(0.82–1.50)
High	1.01	(0.75–1.36)
**Occupational category** ^**a**^		
White-collar	1.00	
Pink-collar	1.08	(0.90–1.29)
Blue-collar	0.94	(0.75–1.19)
**BMI** ^**b**^		
Underweight or Normal (<25)	1.00	
Overweight (≥25.0)	3.03	(2.57–3.57)
**Drinking status**		
No	1.00	
Yes	1.01	(0.85–1.19)
**Walking frequency** ^**c**^		
Inadequate	1.00	
Adequate	0.77	(0.67–0.89)
**Smoking Status**		
Never	1.00	
Former	1.19	(0.93–1.52)
Current	1.52	(1.14–2.03)
**Total kcal intake** ^**d**^		
Inadequate	1.00	
Adequate	1.03	(0.88–1.20)
**Chronic disease** ^**e**^		
No	0.84	(0.68–1.03)
Yes	1.00	
**Survey year**		
2019	1.00	
2020	0.95	(0.80–1.12)
2021	0.94	(0.79–1.11)

*Adjusted for age, marital status, education level, household income, occupational category, BMI, drinking status, walking frequency, smoking status, total kcal intake, chronic disease, and survey year.

^a^Occupational categories were classified into white-collar, pink-collar, and blue-collar groups based on the International Standard Classification of Occupations codes. The “inoccupation” group includes homemakers.

^b^BMI: body mass index; obesity status was defined according to the 2018 Clinical Practice Guidelines for Overweight and Obesity in Korea.

^c^Walking frequency: determined based on the recommended walking activity in the Korean Physical Activity Guidelines.

^d^Total kcal intake was calculated as (carbohydrate (g) × 4 kcal/g) + (protein (g) × 4 kcal/g) + (fat (g) × 9 kcal/g).

^e^Chronic disease: included diagnosed diseases such as hypertension and dyslipidemia.

Table 3 presents the subgroup analysis of the relationship between the work type and insulin resistance, stratified by age, occupational category, BMI, drinking status, walking frequency, and smoking status. The association between shift work and insulin resistance was the strongest among participants aged 40–49 years (OR [95% CI] by age group: 19–29, 1.16 [0.76–1.76]; 30–39, 1.37 [0.79–2.37]; 40–49, 1.88 [1.27–2.76]; 50–59, 1.11 [0.77–1.60]; 60–64, 0.97 [0.54–1.73];), those in pink-collar occupations (white-collar, 1.17 [0.88–1.56]; pink-collar, 1.37 [1.00–1.86]; blue-collar, 1.59 [0.94–2.67]), overweight individuals (underweight/normal, 1.18 [0.94–1.47]; overweight, 1.79 [1.17–2.74]), drinking status (no, 1.27 [0.86–1.87]; yes, 1.29 [1.04–1.61]), and participants reporting inadequate walking frequency (inadequate, 1.34 [1.02–1.78]; adequate, 1.27 [0.98–1.65]). The strongest association was observed among former smokers (never, 1.23 [1.00–1.51]; former, 2.58 [1.28–5.19]; current, 1.17 [0.55–2.47]).

**Table 3:** Subgroup analyses stratified by independent variables.

Variables	TyG index (≥8.5)		
	Work type		
	Day work	Shift work	
	OR	OR^*^	95% CI
**Age (years)**			
19–29	1.00	1.16	(0.76-1.76)
30–39	1.00	1.37	(0.79-2.37)
40–49	1.00	1.88	(1.27-2.76)
50–59	1.00	1.11	(0.77-1.60)
60–64	1.00	0.97	(0.54-1.73)
**Occupational category** ^**a**^			
White-collar	1.00	1.17	(0.88-1.56)
Pink-collar	1.00	1.37	(1.00-1.86)
Blue-collar	1.00	1.59	(0.94-2.67)
**BMI** ^**b**^			
Underweight or Normal (<25)	1.00	1.18	(0.94-1.47)
Overweight (≥25.0)	1.00	1.79	(1.17-2.74)
**Drinking status**			
No	1.00	1.27	(0.86-1.87)
Yes	1.00	1.29	(1.04-1.61)
**Walking frequency** ^**c**^			
Inadequate	1.00	1.34	(1.02-1.78)
Adequate	1.00	1.27	(0.98-1.65)
**Smoking Status**			
Never	1.00	1.23	(1.00-1.51)
Former	1.00	2.58	(1.28-5.19)
Current	1.00	1.17	(0.55-2.47)

*Adjusted for all other covariates.

^a^Occupational categories were classified into white-collar, pink-collar, and blue-collar groups based on the International Standard Classification of Occupations codes. The “inoccupation” group includes homemakers.

^b^BMI: body mass index; obesity status was defined according to the 2018 Clinical Practice Guidelines for Overweight and Obesity in Korea.

^c^Walking frequency: determined based on the recommended walking activity in the Korean Physical Activity Guidelines.

## Discussion

This large-scale, population-based study showed that Korean female shift workers had significantly higher TyG index values than day workers, indicating greater insulin resistance. This finding aligns with previous research linking shift work to adverse metabolic outcomes [4,5,31]. The association was particularly pronounced among women in their 40s, those in pink-collar occupations, and individuals exhibiting unhealthy lifestyle patterns—including overweight status, physical inactivity, former smoking, and current alcohol use—suggesting that shift work may interact synergistically with these factors to exacerbate metabolic dysregulation. Notably, the optimal TyG index cutoff identified in this study (8.35) underscores its practical value as a screening tool for insulin resistance in occupational health settings.

A plausible pathway linking shift work to increased insulin resistance involves hormonal dysregulation caused by circadian rhythm disruption. According to Xie, Hu [32], exposure to light during night shifts disturbs the suprachiasmatic nucleus, impairing vasopressin secretion and glucose transporter expression, while also reducing melatonin levels—thereby diminishing insulin secretion and increasing hepatic glucose output. Additionally, sleep deprivation and stress from shift work elevate cortisol secretion, which interferes with insulin signaling. Irregular meal timing and chronic inflammation further contribute to impaired glucose metabolism [33]. These findings are consistent with prior meta-analyses showing increased rates of metabolic syndrome and diabetes among shift workers [4,18].

The association between shift work and insulin resistance may be stronger in women, potentially due to sex-specific hormonal and physiological characteristics. Women tend to be more sensitive to metabolic changes due to their inherently higher baseline adiponectin levels, which decrease with circadian rhythm disruption, thereby increasing insulin resistance [32]. Female shift workers also have a 1.5-fold higher prevalence of menstrual irregularities, often linked to polycystic ovary syndrome—a condition characterized by metabolic syndrome components that can further exacerbate insulin resistance [33,34]. Additionally, women are more susceptible to stress and sleep disturbances, both of which can activate the hypothalamic-pituitary-adrenal axis and elevate cortisol levels, further impairing glucose metabolism [12]. Given these sex-specific susceptibilities, recent studies have shown that the TyG index demonstrates higher predictive accuracy for insulin resistance and metabolic syndrome in women than in men [35,36]. Because it relies solely on fasting glucose and triglyceride levels, the TyG index provides a simple, cost-effective tool for early detection of insulin resistance in clinical and occupational settings. Therefore, its application in routine metabolic health screening is particularly valuable for female shift workers, who might otherwise remain underdiagnosed despite elevated metabolic risk. These findings underscore the need for sex-specific screening strategies and occupational health policies that address women’s unique physiological and psychosocial vulnerabilities.

Subgroup analysis further confirmed that the association between shift work and insulin resistance was the strongest among women in their 40s and those in pink-collar occupations. Previous studies have shown that individuals aged 40–50 years experience a marked decline in shift work tolerance, including increased subjective sleepiness, reduced work performance, and diminished recovery capacity, due to the cumulative effects of stress, sleep deprivation, and irregular lifestyle patterns associated with shift work [12]. In contrast, older workers in this study did not exhibit insulin vulnerability, which can be explained by the “healthy shift work effect,” whereby only healthy individuals remain in long-term shift work [37]. Pink-collar jobs—often characterized by service-based labor, low job control, and high emotional demands—expose workers to greater occupational stress and fatigue. Nurses, who constitute a significant portion of this workforce, frequently report limited opportunities for rest, exercise, and regular meals during shifts, which may contribute to their heightened metabolic risk [21]. Given the observed associations, structural interventions, such as adjusting shift schedules, increasing job autonomy, and implementing flexible work arrangements, could be considered to reduce occupational fatigue and promote long-term metabolic health in this vulnerable group.

In addition, the negative impact of shift work was particularly pronounced in women with unfavorable lifestyles, which may include obesity-related behavior patterns, such as being overweight and physically inactive. Although these factors are independently associated with increased insulin resistance, their effects appear to be amplified when combined with shift work. Previous studies have shown that shift workers are 1.35 times more likely to develop overweight or abdominal obesity, suggesting that shift work may affect not only weight gain but also fat distribution [38]. Furthermore, women generally exhibit lower shift work tolerance than men, with lower levels of physical activity and poorer dietary patterns, such as irregular or nighttime eating, making them more vulnerable to metabolic disturbances and insulin resistance [12]. One potential mechanism involves leptin dysregulation, as circadian misalignment can suppress leptin secretion, reducing satiety and promoting excessive caloric intake [17,39]. These findings suggest that structured weight management and physical activity programs aligned with shift schedules may be beneficial. Circadian-aligned interventions—such as light exposure therapy or melatonin supplementation—may also help restore hormonal balance and improve metabolic outcomes.

A significant association between shift work and alcohol consumption has been consistently reported [40,41], and the present study also found that the relationship between shift work and insulin resistance was particularly pronounced among female drinkers. One plausible explanation is that alcohol serves as a coping mechanism for sleep disturbances caused by shift work [26]. Compared to other sleep-inducing strategies, such as improved sleep hygiene or medication, alcohol is more accessible; however, tolerance can develop quickly, leading to increased consumption and a harmful cycle. According to Jeong, Kong [26], female shift workers exhibit a higher frequency of alcohol use, often as a strategy to manage sleep difficulties and stress. Increased alcohol intake can worsen insulin resistance by impairing liver function, disrupting glucose metabolism, and promoting inflammation [42]. These findings support the consideration of integrated sleep and substance use interventions within occupational health programs. Notably, studies conducted in countries with limited access to alcohol have reported weaker associations between shift work and drinking [38], suggesting that policy-level restrictions on alcohol availability may be effective.

A significant association between shift work and insulin resistance was observed only among former smokers, not current smokers. Smoking is recognized as an independent risk factor for insulin resistance, as it impairs insulin signaling in the liver and muscles, disrupts lipid metabolism, and promotes chronic inflammation and oxidative stress [43]. This finding suggests that metabolic vulnerability may persist even after smoking cessation. Although nicotine’s appetite-suppressing effects may help current smokers maintain body weight, former smokers often experience increased leptin levels and weight gain after quitting, potentially exacerbating insulin resistance [44]. Additionally, in individuals with long-term smoking histories, irreversible atherosclerotic changes may have already compromised skeletal muscle blood flow, thereby limiting the metabolic benefits of smoking cessation [45]. Future research should incorporate more detailed assessments of cumulative smoking exposure, such as pack-years. Furthermore, because women in many Asian countries tend to underreport smoking due to social norms [46], more objective data collection methods, such as biochemical verification, are needed to improve measurement accuracy.

This study has some limitations. First, due to its cross-sectional design, causal inferences between shift work and insulin resistance cannot be definitively established. Future longitudinal cohort studies and quasi-experimental designs are warranted to clarify causality. Second, several key variables were self-reported, which may have introduced recall and social desirability biases. In particular, socially sensitive behaviors such as smoking and alcohol consumption among women are likely underreported. Future research should incorporate objective biomarkers (e.g., blood cotinine for smoking; urinary ethyl-glucuronide for alcohol) and device-based assessments of physical activity and sleep to improve data validity. Third, although this study utilized nationally representative secondary data, it lacked information on dietary patterns, sleep duration and quality, and chronotype (e.g., morningness–eveningness preference), which are well-known determinants of circadian and metabolic health. Although insulin resistance patterns may differ by menopausal status, our analyses focused on a working-age population (19–64 years) and therefore did not account for menopausal status. Future studies should adopt a life-course approach that considers reproductive stages—such as menopause—when examining these associations. Finally, shift work was classified as a binary variable (day vs. shift work), limiting the ability to capture heterogeneity in shift type, rotation pattern, and exposure duration. Future research should apply more refined exposure measures that distinguish among fixed-night, rotating, or split-shift schedules.

Despite these limitations, this study has several notable strengths. First, it utilized a large, nationally representative dataset (KNHANES), which enhances the generalizability of the findings to Korean women in the working population. Second, whereas previous studies have primarily focused on behavioral risk factors, such as smoking or alcohol consumption, this study emphasizes shift work—a structural occupational exposure—as a key contributor to insulin resistance and provides sex-specific insights into workplace-related metabolic risks. Third, the study employed the TyG index, a validated and clinically accessible surrogate marker for insulin resistance, which improves the clinical applicability and public health relevance of the findings. Finally, by focusing exclusively on women aged 19–64 years, the study captures a critical life stage marked by hormonal transitions and active labor force participation. This sex-specific approach addresses a gap in occupational health research and contributes valuable evidence to support targeted metabolic health interventions and workplace policy development for women.

## Conclusion

This study utilized data from the KNHANES to examine the association between shift work and insulin resistance, with a particular focus on metabolic vulnerability among female workers through subgroup analysis. The findings revealed that shift workers had higher odds of insulin resistance than day workers, and this trend was more pronounced among subgroups of female workers with vulnerable lifestyle or occupational characteristics. Notably, the TyG index was employed as an indirect marker of insulin resistance, highlighting its clinical utility as a simple and cost-effective tool, particularly suitable for evaluating metabolic risk in women. Importantly, unlike previous studies that primarily focused on male or gender-neutral populations, this study emphasizes the importance of considering sex-specific physiological and social factors in occupational health research. The findings support the implementation of tailored interventions for female shift workers that incorporate lifestyle components and shift-schedule adjustments aligned with individual circadian rhythms. Such approaches may inform the development of personalized circadian-based strategies to reduce metabolic risk.
